# Catalyst Selection for Body-Temperature Curable Polyurethane Networks from Poly(δ-Decalactone) and Lysine Diisocyanate

**DOI:** 10.3390/polym17182548

**Published:** 2025-09-20

**Authors:** Marine Boursier, Aurelien Lebrun, Karine Parra, Sylvain Caillol, Claire Negrell, Julien Pinaud

**Affiliations:** 1 ICGM, CNRS, ENSCM, Université de Montpellier, Montpellier, Francesylvain.caillol@enscm.fr (S.C.); 2 PAC Chimie Balard Montpellier—UAR2041, CNRS, ENSCM, Université de Montpellier, Montpellier, France; aurelien.lebrun@umontpellier.fr (A.L.); karine.parra1@umontpellier.fr (K.P.)

**Keywords:** biocompatible, polyurethane, poly(δ-decalactone), lysine diisocyanate ethyl-ester, body temperature

## Abstract

With aging, harsh working conditions or sports injuries, the meniscus can degrade, causing pains to the patient. Nowadays, the treatment consists of the surgical replacement of this cartilage. Since this procedure can lead to complications due to open wounds and potential infections, synthesizing a polyurethane-based injectable joint filler represents an interesting alternative. In this study, poly(δ-decalactone)triol oligomers and Lysine diisocyanate were chosen as starting monomers to create an isocyanate-based prepolymer, because of their biocompatibility and liquid state at room temperature. Nevertheless, to fully replace the meniscus, the joint filler must crosslink in vivo, and this should occur in a short time window. Accordingly, in this work, we studied the catalytic activity of a range of relatively safe compounds for the alcohol/isocyanate addition reaction. A preliminary ^1^H NMR kinetic study of the catalyzed addition of 1-butanol or 3-pentanol on lysine diisocyanate ethyl ester at body temperature has been performed to reach this objective. Among catalysts, stannous octoate was the most effective with either primary or secondary alcohol, allowing them to reach 92 and 80% alcohol conversion, respectively. In addition, the conversion of the primary and secondary isocyanates of lysine diisocyanate ethyl ester was monitored for all the catalysts and revealed different behaviors depending on the catalyst employed. Stannous octoate, unlike the others, showed a similar reactivity for primary and secondary isocyanates with conversions of 49 and 47%, respectively. Finally, when employing the most effective catalyst, curing of the poly(δ-decalactone) triisocyanate with glycerol at 35 °C provided a polyurethane elastomer that exhibits an elastic modulus of 519 kPa and a swelling index lower than 3% in PBS, making it suitable for injectable polyurethane joint filler application.

## 1. Introduction

Polyurethane (PU), is the sixth most widely used polymer family, with a global market value of $80.89 billion in 2024, and it is expected to grow 4.3% from 2025 to 2034 [1]. On an industrial scale, polyurethanes are synthesized via the polyaddition between a polyol and a diisocyanate [2]. These polymers are extensively utilized due to their versatility and remarkable properties, which can be tailored due to the broad variety of available polyols. Indeed, thanks to their excellent mechanical and thermal properties and chemical resistance, PUs are an important class of polymeric material, finding applications in various sectors of daily life. Major applications include construction, furniture and the automotive industry [2,3]. Additionally, due to their adhesive properties, PUs are also used as glues and in varnishes and paints [4]. Furthermore, polyurethanes can be made biocompatible [5,6,7]—and even physio-degradable [5,6]—depending on the monomers used, enabling their application in the medical field for products such as catheters, short-term implants, body adhesives for wound dressings (e.g., pressure-sensitive adhesives) and implantable surgical glues [5,8]. This physio-degradability mostly relies on the polyols [9,10]. For instance, polyester-polyols possess ester bonds in the polymer backbone that can break down through hydrolysis in the presence of water and enzymes [10], generating harmless degradation products. In addition, polyester-polyols allow the production of PUs with a wide range of mechanical properties, due to the different polyesters available. For example, PUs derived from polyesters possessing high T_g_ such as polylactic acid (PLA) and poly(lactic-co-glycolic) (PLGA), are hard materials, while those obtained from low T_g_ polyesters (polylactones: PCL, PδDL…) can be rubbery. Among biomedical applications, PUs such as Actifit, a PU made from poly(ε-caprolactone) and a cycloaliphatic diisocyanate, have been used for menisci replacement implants [11,12]. Indeed, the fibrocartilage present in the knees can age and degrade over time or can be torn during injuries, ultimately leading to osteoarthritis and, thus, pain for the patient. As cartilage does not regenerate spontaneously, an arthroscopic (partial) meniscectomy is often performed, followed by a meniscal allograft transplantation of scaffolds such as Actifit [13]. However, these cartilage replacements induce harsh interventions with post-operation pain and possible complications such as rejection and infection. When meniscuses only suffer from tear injury, sutures are the gold standard, but staples, stingers or screws can also be employed [14]. Nevertheless, these surgical procedures are challenging and time consuming. Injectable cartilage fillers or adhesives thus appear as a good solution to overcome the above-mentioned drawbacks of meniscus repairment/replacement surgery [15,16]. For decades, this solution has been documented in the literature, most of the time with the means of hydrogels [16,17,18]. Recently, Bahatibieke et al. [19] and Fan et al. [20] synthesized joint fillers based on polyurethanes. The materials synthesized by Bahatibieke et al. were made with poly(ethylene glycol) (PEG)/polyethylene castor oil/isophorone diisocyanate (IPDI) prepolymers crosslinked either with water, butane diol and/or gelatin. Foams were then obtained within 5–10 min without a catalyst. Fan et al. synthesized linear polyurethanes by adding hexamethylene diisocyanate (HDI) to PEG first, then N-BOC-serinol. The protection group was then removed, leading to PU-bearing pending amino groups, functionalized in turn by kartogenin (inducer of chondrogenic tissue formation). As one may notice, these materials are mainly petroleum-based and may degrade into compounds that could accumulate in the body, such as high molecular PEG [21,22]. Accordingly, we decided to synthesize a polyurethane for application as an injectable cartilage adhesive. In order to develop a polyurethane that was suitable for use in the repair of menisci by injection, poly(δ-decalactone) was selected as the polyester polyol due to its low glass transition temperature (Tg), its biocompatibility and the fact that it remains in a liquid state at room temperature [23,24]. A low Tg ensures that the resulting network remains soft and elastomeric at physiological temperature, providing mechanical compatibility with the native meniscus and preventing brittle behavior. Furthermore, δ-decalactone is a biobased monomer and its ring-opening polymerization enables the synthesis of star-shaped polyols with hydroxyl functionalities of over two, which are suitable for forming crosslinked networks. Various aliphatic diisocyanates, such as HDI [25,26], IPDI [26,27] and lysine diisocyanate ethyl ester (LDI) [26], have been used in biomedical materials for the isocyanate component. Of these, LDI is the most suitable candidate, as it is biobased and degrades into the naturally occurring amino acid L-lysine, offering both biocompatibility and bioresorbability.

Unlike most medical-grade polyurethanes, which are synthesized and processed outside the body before implantation, our approach relies on the in situ polymer network formation within the body. This strategy imposes strict constraints on the formulation: curing must occur rapidly at body temperature (approximately 37 °C) without the need for external heating. Aliphatic isocyanates are more biocompatible but typically exhibit low reactivity towards alcohols under these conditions, especially in the absence of a catalyst [28]. Therefore, it is essential to select monomers with sufficient reactivity at physiological temperature and to identify catalysts with low or no toxicity [29,30,31,32].

Over the past decade, several research teams have been working on finding non-toxic alternative catalysts for the synthesis of polyurethanes [29,30,31,32,33]. For instance, Sardon and his team [34] explored the effect and the mechanisms of Brönsted acids on hexamethylene diisocyanate (HDI) and isophorone diisocyanate (IPDI) with PEG_1500_ at 20 °C. Alsarraf et al. [35] and Arnould et al. [36] studied the influence of basic catalysts on primary and secondary alcohols with HDI and PEG_600_ at 60 °C and PPG at 80 °C, respectively. Arnould et al. [37] also studied the impact of organometallic catalysts on the synthesis of PU with both cycloaliphatic isophorone diisocyanate (IPDI) and aromatic 4,4-methylenebis(phenyl-isocyanate) (4,4-MDI) at 80 °C. Regarding more specifically biomedical applications, Sardon [26] and his team employed DBU as a catalyst for the synthesis of PU at room temperature from several diisocyanates, including HDI, IPDI and LDI. Nevertheless, the PU synthesis took more than 3 h to be completed and the catalyst was removed by purification. Yeoh et al. [27] and Guelcher et al. [38] also explored the use of triethylene diamine (TEDA) as a catalyst for the synthesis of a biocompatible PU. Nevertheless, this catalyst was found to favor the reaction with water over the isocyanate-alcohol reaction, thus leading to foams. On their side, Stevens et al. [25] used DABCO to catalyze the HDI/alcohol reaction in order to produce biocompatible hydrogels, but curing was performed at 85 °C for 24 h. There is thus still the need for a safe catalyst to be able to promote the alcohol/aliphatic isocyanate reaction at room or body temperature. In this work, the reactivity of primary and secondary alcohols towards lysine diisocyanate ethyl ester (LDI) in the presence of different catalysts was kinetically evaluated at 35 °C. To this end, 1-butanol and 3-pentanol were first employed as molecular analogs of primary and secondary alcohol polymer chain-ends. Regarding the catalysts, Brönsted acids, tertiary amines and organometallic compounds already known to promote the isocyanate/alcohol addition reaction and with LD_50,_ over 300 mg/kg (in rats) were targeted. Thus, methane sulfonic acid (MSA), trifluoroacetic acid (TFA), *N*,*N*-dimethylcyclohexylamine (DMCHA) [39] and stannous octoate—Sn(Oct.)_2_ were tested [39,40]. Then, a PδDL-triol was synthesized and functionalized with LDI at 80 °C, in the absence of a catalyst to lead to a PδDL triisocyanate. In the second step, this new prepolymer was then reacted at 35 °C with glycerol and a catalyst to produce a fully biobased polyurethane elastomer that was able to cure at body temperature in a short time [41,42,43,44,45,46].

## 2. Experimental Section

### 2.1. Material

δ-decalactone (δDL—≥ 98%), 1, 1, 1-tris(hydroxymethyl)propane (trimethylol propane—TMP—≥ 98%), glycerol (≥99.5%), tin(II) bis(2-ethylhexanoate) (stannous octoate, Sn(Oct.)_2_—92.5–100%), 1, 3, 5-trioxane (>99%) and 3-pentanol (98%) were purchased from Sigma Aldrich (Darmstadt, Germany). P-toluenesulfonic acid monohydrate (pTSA—>98%), methane sulfonic acid (MSA—>99%) and 1-butanol (>99%) were purchased from TCI Europe N.V (Zwijndrecht, Belgium). l-Lysine diisocyanate ethyl ester (LDI—97%), trifluoro acetic acid (TFA—99%, extra pure), Ambelyst^TM^ 15H-dry, di-n-butylamine (99%) and PBS tablets were purchased from Thermo Fischer Scientific (Geel, Belgium). Dimethylformamide (DMF) was purchased from VWR (Radnor, PA, USA). Toluene (>99.7%) and hydrochloric acid (>37%) were purchased from Honeywell (Saint Priest, France). Chloroform and anhydrous absolute ethanol were purchased from Carlo Erba (Val-de-Reuil, France). CDCl_3_ was provided by Eurisotop (Saint-Aubin, France). Except for δ-DL, which was purified by distillation under reduced pressure (120 °C at 0.3 mBar), all the reagents were used as received.

A speed mixer^®^ (DAC 400.2 VAC-P, Hauschild, Hamm, Germany) was used to homogeneously mix the formulations.

### 2.2. Kinetic Study

To choose the best operating conditions for reactions between lysine diisocyanate and a polyol, kinetic studies were first performed on model reactions with 1-butanol or 3-pentanol, and LDI with several catalysts.

Table 1 displays the required volume of each reagent to reach the concentration of 1 M for the isocyanate (LDI), 2 M for the alcohol (1-butanol or 3-pentanol) and 0.01 M for the catalyst (TFA, MSA, DMCHA and Sn(Oct.)_2_).

As a representative example, for the kinetic study of experiment 3 in Table 1, (LDI:butanol—1:2 + 0.01 M of TFA), 117 μL of LDI, 111 μL of 1-butanol, 20 μL of the TFA solution (0.31 M in CDCl_3_), about 10 mg of 1, 3, 5-trioxane and deuterated chloroform (*q.s.*, 610 μL) were introduced into a NMR tube. ^1^H NMR spectra were recorded at t = 0 s and then every 5 min for one hour.

The turnover frequency (TOF) is calculated with the following formula:

(1) $$\mathrm{TOF}=\frac{number\;of\;moles\;of\;product\;formed}{number\;of\;moles\;of\;catalyst\times time}=\;\frac{rate\;of\;product\;formation}{catalyst\;concentration}$$

where the rate of the product formation is the slope of the linear part of the “conversion vs. time” curve.

### 2.3. General Procedure for the Synthesis of PδDL Triol Oligomers

The following is a representative example of the general procedure we used to synthesize a poly(δ-decalactone) triol, PδDL, with a degree of polymerization of 15 units ($\bar{Dp}$_n_ = 15, PδDL_15-3OH_). In a clean and dry Schlenk equipped with a stir bar, trimethylolpropane (5.26 g, 39.2 mmol) and δ-decalactone (100 g, 588.1 mmol) were combined and magnetically stirred under vacuum/ nitrogen flux cycles (alternation of 10 min each, 5 times). After the TMP’s complete dissolution and the end of the cycles, the catalyst Amberlyst^TM^ 15H (25.85 g, 82.3 mmol) was added under nitrogen flux. The reactor was closed by a septum, and the mixture was allowed to react for 8 days at room temperature, until 70% monomer conversion was reached. Then, Amberlyst^TM^ was removed by simple filtration, and the PδDL_15-3OH_ was used without purification.

### 2.4. Functionalization of the Triol Oligomer into Isocyanate

Poly(δ-decalactone) triisocyanate (PδDL_15-3NCO_) was synthesized in bulk by functionalizing the previously synthesized PδDL_15-3OH_. The following is a representative example of our general procedure when synthesizing PδDL_15-3NCO_. Four molar equivalents of lysine diisocyanate ethyl ester (LDI—6.23 g, 26.27 mmol) were placed in a round-bottom flask equipped with a stir bar under a nitrogen atmosphere and heated at 80 °C under agitation. Then, PδDL_15-OH_ (22.53 g, 6.57 mmol), placed in a syringe of 50 mL equipped with a needle (gauge 16), was added dropwise into the round-bottom flask over three hours with the help of a syringe pump. The reaction was allowed to continue for an extra hour at 80 °C before switching off the heating. After cooling to room temperature, the reaction mixture was flushed with nitrogen to remove any residual moisture and ensure the stability of the isocyanate groups. The final product was transferred to a sealed container and stored in a fridge to prevent potential side reactions or dimerization of the isocyanate groups. No purification steps were required, and the polymer was recovered quantitatively with a mass of 28.76 g, corresponding to a 100% yield.

### 2.5. Synthesis of the Elastomeric Joint

In a polypropylene container, PδDL_15-3NCO_ (5 g, 2.61 mmol), glycerol (0.20 g, 2.18 mmol) and Sn(Oct.)_2_ (3%_mol_ vs. isocyanate—25.4 μL, 0.08 mmol) were added and mixed vigorously with a speed mixer^®^ for 2 min at 2500 rpm. The mixture was then poured into a mold and allowed to crosslink at 35 °C in an oven overnight.

### 2.6. Nuclear Magnetic Resonance (NMR)

The Nuclear Magnetic Resonance (NMR) spectra were recorded on a Bruker Avance III 400 MHz spectrometer. The instrumental parameters for recording ^1^H NMR spectra were as follows: flip angle 30°, acquisition time 18 s, pulse delay 1 s, number of scans 8, and pulse width 5 μs. NMR spectra were recorded in deuterated chloroform (CDCl_3_) at 35 °C. CDCl_3_ served as an internal standard (δ = 7.26 ppm).

### 2.7. Size Exclusion Chromatography (SEC)

Average molar masses ($\bar{Mn}$) and dispersities (Ð) of polymers were determined by size exclusion chromatography (SEC) analyses, which were conducted on a system composed of an Agilent Infinity I for pump degasser, oven and detector 1260 VWD G1314F and a Varian 390-LC Multi detector suite, fitted with differential refractive index, light scattering, and viscosimeter (Agilent, Santa Clara, CA, USA). The system was equipped with a guard column (Agilent Mesopore, 50 × 7.5 mm, Agilent, Santa Clara, CA, USA) and two columns (Agilent Mesopore and Resipore, 300 × 7.5 mm, Agilent, Santa Clara, CA, USA). The mobile phase was tetrahydrofuran (THF) at a flow rate of 1.0 mL·min^−1^. Toluene was added to the samples as a flow marker and samples (typical concentration: 5 mg·mL^−1^) were filtered before analysis (0.20 μm PTFE filter). Poly(methyl methacrylate) (PMMA) standards (550–2,210,000 g·mol^−1^) were used to determine the calibration curve.

### 2.8. PδDL Triol HEW Determination

In order to determine the hydroxyl equivalent weight (HEW) of the PδDL_15-3OH_, 3 samples were prepared with ca., 50 mg of the polyol and 20 mg of 1,3,5-trioxane in 1 mL of CDCl_3_. These mixtures were added into 3 NMR tubes and ^1^H spectra were recorded. The peak of trioxane was integrated from 5.25 to 5.67 ppm and was calibrated as 1000 equivalents. The integration of the signal corresponding to the proton CH-OH (g″ in S.I. 1) of the PδDL_triol_, at 3.62 ppm was then measured and the HEW was calculated as follows:

(2) $$HEW=\;\frac{n_{H}\;polyol*Int.\;triox*\;m_{polyol}\;}{n_{H}\;triox*Int.\;polyol*\frac{m_{triox}}{M_{triox}}}$$

where n_H_ is the number of hydrogens integrated into each species; here 1 for the polyol and 6 for the trioxane, Int. are the integrals of these hydrogens, m_polyol_ and m_triox_ are the mass (in g) of the polyol and the trioxane (with M_triox_ = 90.08 g·mol^−1^).

The HEW of this sample is 1143.3 g·mol^−1^.

### 2.9. PδDL Triisocyanate IEW Determination

The isocyanate equivalent weight (IEW) was determined thanks to a back titration. A certain amount of PδDL_15-3NCO_ (ca., 500 mg) was placed in a round-bottom flask equipped with a stir bar, then 25 mL of a di-n-butylamine 0.5 M in toluene solution were subsequently added and the solution was placed under agitation for 2 h. Afterward, 30 mL of ethanol and a few drops of green bromocresol were added. This solution was stirred vigorously. Finally, the amine excess was titrated by a hydrochloric acid solution at 0.1 M. When the solution turned from blue to yellow and stayed so for more than 60 s, the volume was written down as V1. The same procedure was reproduced in the absence of PδDL_3NCO_ to determine V0.

IEW was calculated as follows:

(3) $$IEW=\;\frac{m_{P\delta DL-NCO}}{\left(V0-V1\right)*C_{HCl}\;}$$

with m_PδDL-NCO_, the mass of PδDL_15-3NCO_ (in g, accurate to 0.1 mg), V0 and V1, the volume (in L) of the blank and the control samples, and C_HCl_ the concentration of HCl (in mol·L^−1^).

### 2.10. Viscosity and Gelation Time Determination

Rheology measurements were performed on an Anton Paar MCR 302 rheometer (Anton Paar, Graaz, Austria). The gelation times were measured with a disposable plan-plan shaft of 25 mm in diameter. They were determined by the crossover of the storage modulus (G’) and the loss modulus (G″) during an oscillatory experiment at 1 Hz, 35 °C and 2% of deformation, according to the previously determined linear domain.

### 2.11. Swelling Index (SI) and Gel Content (GC)

Three samples from the same material (ca., 110 mg) were immersed individually into 1 mL of DMF at room temperature, and three others in PBS at 37 °C and all were allowed to swell for 24 h. The swelling index (SI) was calculated as follows:

(4) $$SI=\frac{m_{2}-m_{1}}{m_{1}}*100$$

where m_1_ is the initial mass and m_2_ is the mass of the swollen material. SI is the average of the values for the 3 samples.

After the measurement of SI, the 3 samples were dried at 80 °C in a vacuum oven for 24 h. The gel content (GC) was calculated as follows:

(5) $$GC=\frac{m_{3}}{m_{1}}*100$$

where m_3_ is the mass of the dry sample. GC value is the average of the three measurements.

### 2.12. Thermogravimetric Analyses (TGA)

Thermogravimetric analyses were conducted by utilizing a Netzsch TG 209F1 apparatus (Netzsch, Selb, Germany). Around 10 mg of the sample were loaded into an alumina crucible and heated from room temperature to 550 °C at a rate of 20 °C·min^−1^ under a nitrogen atmosphere (40 mL·min^−1^).

### 2.13. Differential Scanning Calorimetry (DSC)

Differential Scanning Calorimetry (DSC) analyses were performed utilizing a Netzsch DSC200F3 Maia calorimeter (Netzsch, Selb, Germany), calibrated with indium, n-octadecane, and n-octane standards. Nitrogen served as the purging gas. About 10 mg of the sample were positioned in a perforated aluminum pan, and the thermal properties were recorded within the range of −100 °C to 90 °C at a rate of 20 °C·min^−1^ to observe the glass transition temperature. The T_g_ values were determined during the second heating ramp to remove the polymer’s thermal history.

### 2.14. Fourier Transform Infrared Spectroscopy (FTIR)

Infrared analyses were conducted using a Nicolet 210 Fourier Transform Infrared (FTIR) Spectrometer (Thermo Fisher Scientific Inc., Waltham, MA, USA) with an ATR accessory. The specific IR absorptions mentioned in the text are listed in cm^−1^.

### 2.15. Shore A Hardness

The Shore A hardness of the material was measured at room temperature using a Sauter HDD 100-1 Shore A durometer (Sauter, Bale, Suisse). This durometer has a maximum reading of 100 ShA and a precision of 0.1 ShA. The reported values are the averages of five consecutive measurements, along with the standard deviation.

### 2.16. Tensile Test

Tensile tests were carried out using an Instron 3366L5885 mechanical tester fitted with a 100 N load cell. Flat specimen samples were fabricated thanks to a Teflon mold, and their dimensions were measured using a micrometer (l_x_w_x_t = 37.4_x_6.6_x_2.0 mm) before conducting the tests. The tensile speed was set to 5 mm·s^−1^. Each result is reported as the mean ± standard deviation (*n* = 5). Young’s modulus was determined as the slope of the linear region (from 0 to 5% deformation).

## 3. Results and Discussion

### 3.1. Kinetic Study

**Scheme 1:**
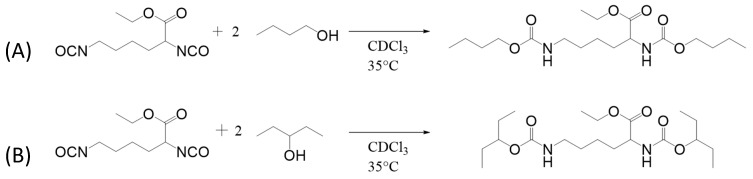
Reactions of LDI with BuOH (**A**) and PentOH (**B**).

As outlined in the literature, the majority of polyurethanes employed in biomedical applications are synthesized ex vivo, usually at elevated temperatures (e.g., 80 °C), before being implanted once fully cured. In contrast, our approach aims to produce the polyurethane network in situ, i.e., directly within the body. This strategy imposes several critical constraints: the polymerization must proceed efficiently at body temperature (approximately 37 °C), which rules out conventional thermal curing. It also restricts the choice of monomers to those with sufficient reactivity under mild conditions, and requires the use of catalysts that are both effective and with low or no toxicity. To evaluate the efficiency of catalysts at body temperature, kinetic studies were thus carried out with model molecules in the presence of different catalysts of low toxicity (Table 2). To this end, the alcohol and isocyanate compounds and an internal standard (1,3,5-trioxane) were added to an NMR tube with CDCl_3_, to determine the kinetics of the isocyanate-alcohol addition reaction at 35 °C. Two model couples of molecules were chosen: lysine diisocyanate (LDI), containing primary and secondary isocyanates, with 1-butanol (Scheme 1A) or 3-pentanol (Scheme 1B), in order to evaluate the reactivity of primary and secondary alcohols towards the two different isocyanates of LDI. Monofunctional alcohols were chosen to keep a simple system and allow the solubility of the final product. Reactions were performed using an OH:NCO stoichiometric ratio of 1:1, with a concentration of alcohol, LDI and a catalyst of 2/1/0.01 mol·L^−1^, respectively.

The different experiments, as well as the conversions obtained after 60 min of reaction and the turnover frequency (TOF) of each catalyst, are reported in Table 3. At first, control reactions were performed with LDI and the primary alcohol (exp. 1, Table 3) or the secondary alcohol (exp. 2, Table 3) without a catalyst, in order to determine their intrinsic kinetics. Then, TFA, MSA, DMCHA and Sn(Oct.)_2_ were evaluated for both couples (exp. 3–10, Table 3).

**Figure 1:**
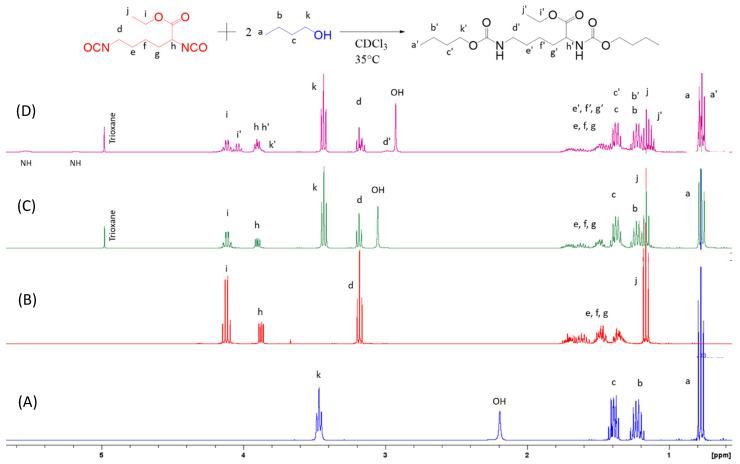
^1^H NMR spectra and assignments of BuOH (blue-(**A**)), LDI (red-(**B**)), reaction media corresponding to exp. 5, Table 3 at t = 0 (green-(**C**)) and t = 1 h (purple-(**D**)) (CDCl_3_, 400 MHz, 35 °C).

**Figure 2:**
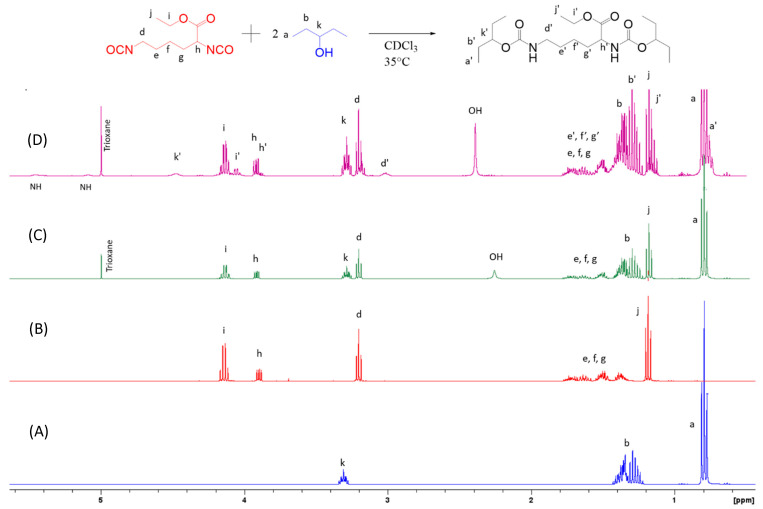
^1^H NMR spectra and assignments of PentOH (blue-(**A**)), LDI (red-(**B**)), reaction media corresponding to exp. 6, Table 3 at t = 0 (green-(**C**)) and t = 1 h (purple-(**D**)) (CDCl_3_, 400 MHz, 35 °C).

The ^1^H NMR spectra with the assignments of the signals from the reactants and the urethane adducts are depicted in Figure 1 and Figure 2, for the reactions with 1-butanol and 3-pentanol, respectively. For instance, Figure 1 represents the stacking of the spectra of the 1-butanol (A), LDI (B) and the reaction media at t = 0 (C) and t = 1 h (D) in the presence of MSA as a catalyst (exp. 5—Table 3). These spectra were used to monitor the conversion of the primary isocyanate and the alcohol. The signal of 1,3,5-trioxane (internal standard) at 4.95 ppm was used as a standard and calibrated to 6H in each spectrum. Then, the signals corresponding to the protons of interest were integrated and their integrals were used to calculate the conversion with the following formula:

(6) $$Conversion\left(\%\right)=1-\frac{{Int.}_{t=x}}{{Int.}_{t=0}}*100$$

where Int._t=x_ and Int._t=0_ are the values of the integrals of the signals of the protons of interest (i.e., the proton in α-position to the monitored function), at t = x and t = 0.

Thus, for the primary isocyanate, the conversion was followed thanks to both the diminution of the signal corresponding to the methylene protons in α to the isocyanate (proton *d*, C**H_2_**-NCO) at 3.18 ppm and by monitoring the increase in the signal corresponding to the protons in α of the primary urethane (proton *d*′, C**H_2_**-NH(CO)O) at 3.0 ppm. For the alcohol, the conversion was determined by using integration of the signal attributed to the protons in α to the alcohol (proton *k*, C**H_2_**-OH) at 3.44 ppm. Unfortunately, the disappearance of the signal corresponding to the proton in α to the secondary isocyanate (proton *h*, C**H**-NCO) at 3.9 ppm cannot be monitored because the signal attributed to the proton in α to the secondary urethane (proton *h*′, C**H**-NH(CO)O) appears with the same chemical shift. For this reason, the conversion of the secondary isocyanate was considered to be the difference between conversions of the alcohol and the primary isocyanate (Equation (7)).

(7) $$Conversion\;Iso\;II\left(\%\right)={Conv.}_{alcohol}-{Conv.}_{IsoI}$$

The same procedure was used to calculate the conversion for the reaction with LDI and 3-pentanol. In this case, the proton in α to the alcohol (proton *k*, C**H**-OH) was at 3.29 ppm. In addition, for this couple, the conversion of the alcohol was also confirmed by the appearance of the protons near the urethane (proton *k*′, C**H**-O(CO)) at 4.48 ppm, which is not isolable for LDI + 1-butanol.

The same procedure was followed for all the kinetic studies, the catalyst’s nature being the only parameter changed.

**Figure 3:**
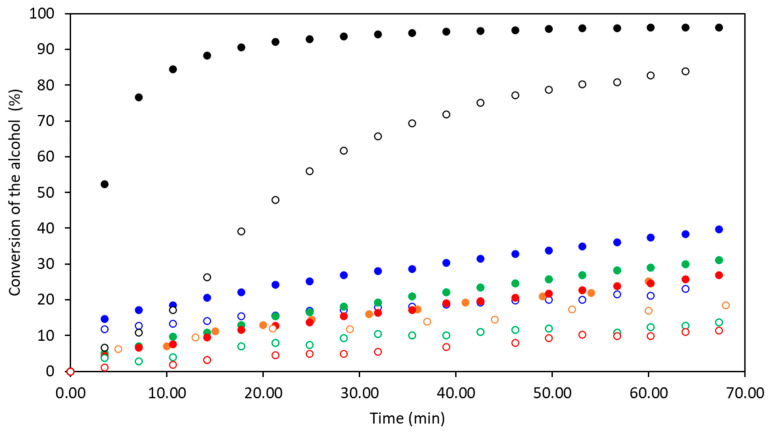
Conversion of alcohol functions with time during the reaction of BuOH or PentOH with LDI in the absence or in the presence of catalysts; for BuOH − control (exp-1, orange ●), BuOH + TFA (exp-3, green ●), BuOH + MSA (exp-5, blue ●), BuOH + DMCHA (exp-7, red ●), BuOH + Sn(Oct.)_2_ (exp-9, black ●), PentOH − control (exp-2, orange ○), PentOH + TFA (exp-4, green ○), PentOH + MSA (exp-6, blue ○), PentOH + DMCHA (exp-8, red ○) and PentOH + Sn(Oct.)_2_ (exp-10, black ○).

**Scheme 2:**
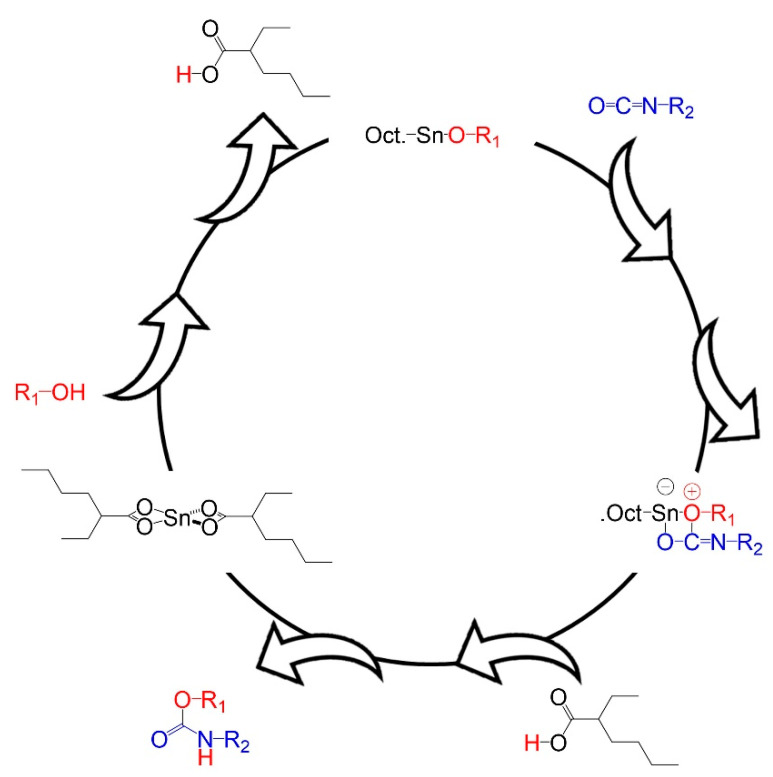
Catalytic cycle of stannous octoate for the isocyanate alcohol addition reaction.

Figure 3 exhibits the evolution of the conversions of 1-butanol and 3-pentanol into urethane functions for each experiment from Table 3 over an hour. First, it can be observed that the conversions of the primary alcohol for all experiments are faster than the ones of the secondary alcohol in similar experimental conditions. Consequently, none of the catalysts tested in this study allow the secondary alcohol to react faster than the primary alcohol. Then, it can be identified that the conversions of the control reactions are very slow, exhibiting 25% and 17% conversion after an hour for BuOH and PentOH, respectively. For the reaction with 1-butanol catalyzed with TFA and MSA at the same concentration (0.5%mol relative to the reactive alcohol function), conversions are slightly higher than the control reaction, reaching almost 30% and 40%, respectively. For the reaction with 3-pentanol, the conversions display almost the same values as the control reaction that was devoid of a catalyst (14% and 22%). The impact of these acids on the reaction catalysis is, thus, moderate. On the other hand, the difference in reactivity between the two alcohols is still noticeable. Among the four catalysts evaluated, the most efficient catalyst is the stannous octoate, which reaches 50% conversion in less than 5 min for 1-butanol and in less than 20 min for 3-pentanol. After one hour, the conversion for 1-butanol is almost complete (>95%) and for 3-pentanol, higher than 70%. On its side, DMCHA seems to have no influence on the reaction kinetics. Indeed, for 1-butanol, the same conversion as the control was obtained (25%) after 1h, while in the same period, only 9% of conversion was obtained with 3-pentanol, showing a counterproductive effect. These results are corroborated by the turnover frequencies (TOF) that remain in the lower tenths for all reactions except for those conducted with Sn(Oct.)_2_ as a catalyst, which displays a TOF of 1083 s^−1^ with 1-butanol and a TOF of 224 s^−1^ with 3-pentanol. Stannous octoate is a commonly used catalyst in urethane reactions and is particularly effective in promoting the reaction between isocyanate and alcohol. The lower steric hindrance and higher reactivity of butanol compared to pentanol allows Sn(Oct.)_2_ to achieve a high conversion rate in a short time. The difference in kinetics between 1-butanol and 3-pentanol in the presence of Sn(Oct.)_2_ can be explained by the mechanism taking place with this catalyst (Scheme 2) [55]. Indeed, although similar pKa has been reported for 3-pentanol and 1-butanol (pKa = 16) [56], Sn(Oct.)_2_ is a bulky molecule; hence, it may be difficult for it to bond to the secondary alcohol that is also hindered. Then, in the second step of the reaction, it can also be more difficult for the ‘stannous—pentanol intermediate’ to bond to the isocyanate due to the steric hindrance of both alcoholates. Transition metals can act as active sites to promote the formation of chemical bonds, and they also lead to better selectivity by stabilizing intermediates (thus minimizing side reactions) [36,37].

**Scheme 3:**
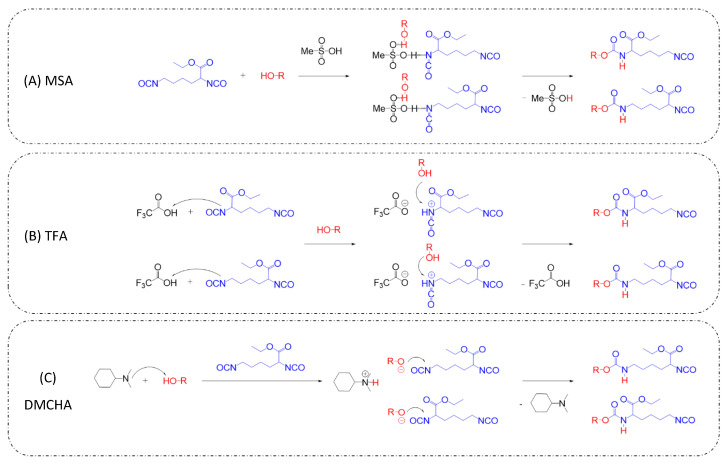
Mechanisms of the activation of the isocyanate, the alcohol, or both, with MSA (**A**) [34,57], TFA (**B**) [34] and DMCHA (**C**).

Acids like TFA and MSA also catalyze the reaction, but with a moderated effect. Thus, this activation is less efficient than that of Sn(Oct.)_2_, which directly coordinates with the oxygen of the isocyanate. TFA and MSA show lower and similar conversions for both alcohols, likely because the activation mechanism proceeds by direct activation of the isocyanate, thus making it less dependent on the structural differences between the alcohols (Scheme 3). In addition, Sardon et al. have shown that TFA did not affect the primary isocyanate of HDI [34]. On its side, DMCHA does not appear to be effective in the reaction of LDI with 1-butanol, nor with 3-pentanol, while it has been reported to have an effective catalyst for other isocyanate/alcohol reactions [39]. Its inability to significantly activate the reaction may be attributed to the presence of residual carboxylic acid functions in LDI. Nevertheless, despite our efforts to demonstrate their presence, no carboxylic acid functions could be detected in LDI.

**Figure 4:**
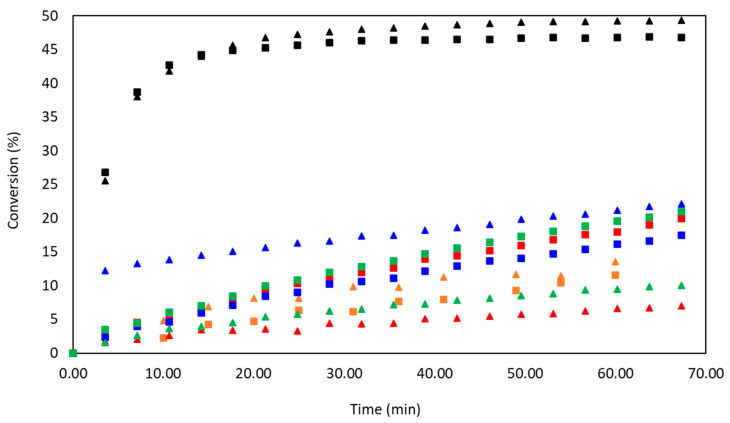
Conversion of the primary (▲) and secondary (■) isocyanates of LDI vs. time with BuOH: Iso I − control (orange ▲), Iso I + TFA (green ▲), Iso I + MSA (blue ▲), Iso I + DMCHA (red ▲), Iso I + Sn(Oct)_2_ (black ▲), Iso II − control (orange ■), Iso II + TFA (green ■), Iso II + MSA (blue ■), Iso II + DMCHA (red ■) and Iso II + Sn(Oct)_2_ (black ■).

Figure 4 and Figure S2 separately depict the conversions of primary and secondary isocyanates with 1-butanol and 3-pentanol, respectively. The conversion values of the reactions after one hour are gathered in Table 3. It can be seen that neither the control sample (e.g., 1-butanol, iso_I_-iso_II_: 14–12%) nor Sn(Oct.)_2_ (e.g., 1-butanol, iso_I_-iso_II_: 49–47%) favor an isocyanate over the other, leading to equi-reactivity of both isocyanates. MSA, on its side, tends to slightly favor the activation of the primary isocyanate (e.g., 1-butanol, iso_I_-iso_II_: 21–16%). Despite the lack of effect on the kinetic rate under these conditions for TFA and DMCHA, it seems that the reactivity of the isocyanates observed is the opposite for the control and the others. As a matter of fact, they promote the reactivity of the secondary isocyanate over the primary one. This can be explained by the fact that the ester group has mesomeric and inductive effects that increase the electrophilicity of the secondary isocyanate. To verify this hypothesis, a kinetic study was performed on the couple IPDI/BuOH, uncatalyzed and catalyzed by TFA or MSA (Figure S3). Indeed, IPDI has primary and secondary isocyanates but is devoid of an electron-withdrawing group. Figure S4 depicts that, such as for LDI, MSA has a better catalytic effect than TFA. Their TOF are 663 s^−1^ and 23 s^−1^, respectively. On the other hand, as in the reference and the reaction with MSA, the IPDI reaction catalyzed by TFA exhibits a higher activation of the primary isocyanate over the secondary one (Figure S5). This confirms that the steric hindrance does not play a role here, but the nature of the side group does.

Accordingly, stannous octoate appears to be the most active catalyst for the alcohol/LDI reaction at 35 °C, particularly with primary alcohols where it allows reaching 90% conversion in a 20 min reaction without favoring one or the other isocyanate. Consequently, the reaction between LDI and a primary alcohol in the presence of stannous octoate was selected for the final cross-linking reaction, leading to the biocompatible injectable adhesive, and was supposed to occur in vivo. Since PδDL displays secondary alcohols as chain-ends, we thus decided to prepare the PU material in two steps: (i) synthesis of an isocyanate-terminated PδDL by reaction of PδDL triol, with an average polymerization degree of 15 (PδDL_15-3OH_), with lysine diisocyanate ethyl-ester (LDI) at 80 °C and (ii) formulation and crosslinking of synthesized PδDL_15-3NCO_ with glycerol in the presence of stannous octoate (reaction occurring in vivo).

**Figure 5:**
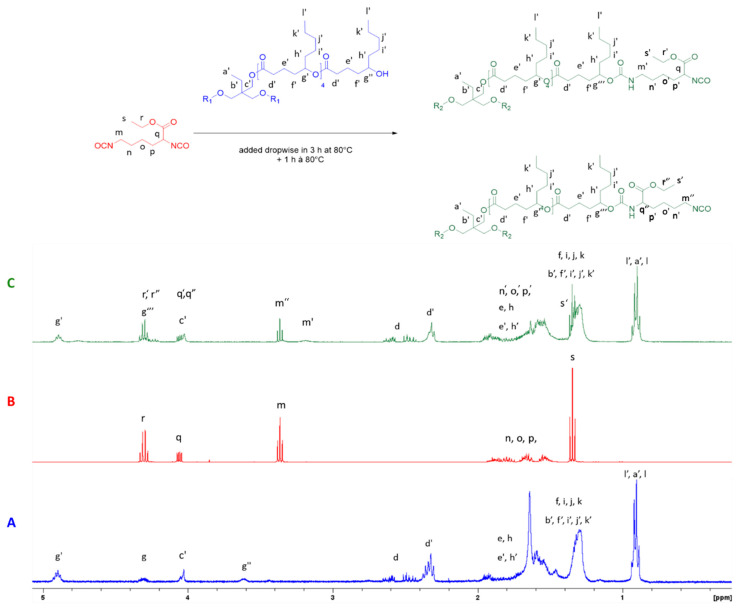
^1^H NMR spectra and assignments of PδDL15-3OH (blue—**A**), LDI (red—**B**) and PδDL15-3NCO (green—**C**) in CDCl_3_.

To this end, PδDL15-3OH was prepared by ring-opening polymerization of δ-decalactone using trimethylolpropane as the initiator, catalyzed by Amberlyst^TM^ 15-H under mild conditions. The resulting triol was subsequently functionalized with four equivalents of LDI at 80 °C, leading to the formation of PδDL15-NCO. To confirm its structure, as prepared, PδDL15-NCO was characterized by ^1^H NMR and FTIR spectroscopy, for which corresponding spectra are depicted in Figure 5 and Figure S6, respectively. As it can be observed in Figure 5, consumption of the alcohol by reaction with the isocyanate of LDI was confirmed by the disappearance of proton *g*″ at 3.62 ppm corresponding to the hydrogen in α position to the alcohol (C**H**-OH). The creation of the urethane bond can be corroborated thanks to the appearance of protons *m*′ at 3.21 ppm (C**H_2_**-NH-C=O), *q*′ at 4.09 ppm (C**H**-NH-C=O), *g*^3^ at 4.36 ppm (C**H**-O-C=O). These values are verified by FTIR (Figure S6). Indeed, the disappearance of the IR bands at 1110 and 3543 cm^−1^_,_ corresponding to the valence of the **C**-OH of secondary alcohol and the valence of the hydroxyl group, as well as the appearance of the IR bands at 1545 (ν N-H_ureth_), 1620 (ν C-N_urea_), 1742 cm^−1^ (ν C=O_urethane_) and 3333 cm^−1^ (ν N-H_ureth_) corresponding to the urethane bond, confirm the reaction of the alcohol with the isocyanate leading to a carbamate [58,59].

As synthesized, PδDL_15-3NCO_ possessed an isocyanate equivalent weight (IEW) of 744 g·eq^−1^, close to the theoretical value (IEW_theo_ = 876 g·eq^−1^), indicating that few oligomerizations occurred during functionalization. This was confirmed by steric exclusion chromatography measurements, since its average number molar mass Mn = 6000 g·mol^−1^ (Ð = 2.1) was slightly higher than the Mn value of the starting PδDL_15-3OH_ (Mn = 4000 g.mol^−1^; Ð = 1.3). With the excess of LDI used during the synthesis of PδDL_15-3NCO_, the average functionality of this isocyanate product is 2.5. Additionally, it displays a viscosity of 24 Pa·s, which is adequate for an application with a syringe and a needle.

### 3.2. Synthesis of the Elastomer

**Scheme 4:**
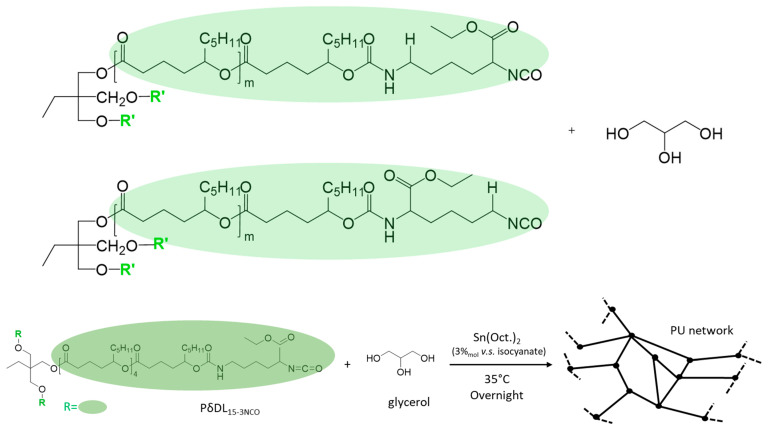
Reaction of glycerol and triisocyanate prepolymer to obtain biocompatible PU elastomer.

**Figure 6:**
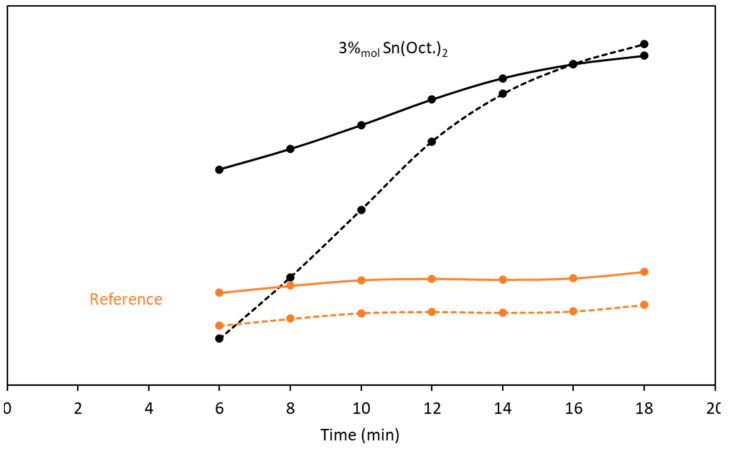
Storage modulus—G’ (interrupted line) and loss modulus—G’’ (continuous line) curves (f = 1 Hz, γ = 0.1%) of a control formulation without a catalyst (orange) and a formulation containing 3% _mol vs. Iso_ of Sn(Oct.)_2_ (black). The rheometer was set up at 35 °C.

**Figure 7:**
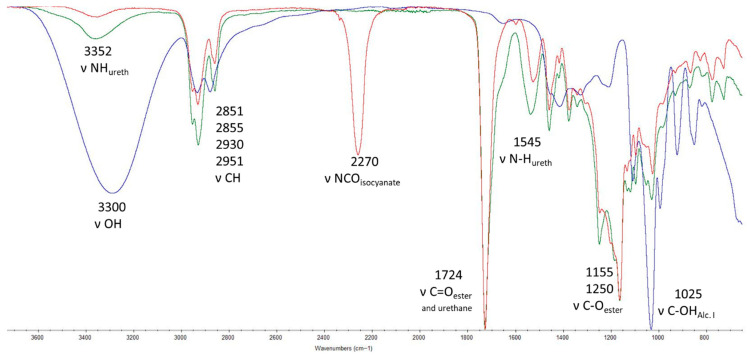
FTIR spectra of PδDL_15-3NCO_ (red), glycerol (blue) and the crosslinked polyurethane (green).

To demonstrate the potential of stannous octoate as a catalyst for the synthesis of the body-temperature curable polyurethane joint, PδDL_15-3NCO_ was reacted with glycerol in the presence of Sn(Oct.)_2_ (Scheme 4). Glycerol was used in this study because it is a biobased triol already present in the body and liquid at room temperature, which possesses two primary alcohols and one secondary alcohol. The material synthesis was performed in bulk at 35 °C, with a OH:NCO molar ratio of 1:1. Gel time of the formulation at 35 °C was then determined by rheology to confirm the fast kinetics of the cross-linking reaction. As expected, the formulation with 3%_mol vs. iso_ of Sn(Oct.)_2_ showed a gel time of 18 min (Figure 6). This is much faster than the control sample, devoid of a catalyst, whose gel time was not reached even after 48 h under the same conditions. Complete curing of the elastomer was achieved overnight at 35 °C, as attested by FTIR analysis (Figure 7), as well as swelling index and gel content measurements. Indeed, no band at 2770 cm^−1^ corresponding to the stretching of the NCO was observed in the FTIR spectra of the final PU material. Secondly, to confirm the complete crosslinking of the network, the gel content (GC) was measured after soaking the material for 24 h in DMF at room temperature, then 24 h at 80 °C under vacuum. A GC of 87% in DMF, consistent with the 20% of remaining δDL monomer left in the PδDL_15-3NCO,_ was measured, thus attesting to the good crosslinking of the network. In order to check the hydrophilicity of the material and its water uptake capacity, the swelling index (SI) was measured after soaking the material for 24 h in PBS at 35 °C. The SI, lower than 3%, shows that the material did not swell in PBS, similar conditions to physiological ones, which is an expected feature for a biomedical device of that kind. The hydrophobic character of the material was also confirmed by contact angle measurements. Indeed, when the PU material was coated on a glass slide, the contact angle of the glass slide rose from 49.7° before coating to 80° (Figure S7). Finally, the mechanical properties of the elastomer were evaluated by tensile strength (Figure S8). Young’s modulus was determined to be 519.4 ± 45.8 kPa, which is much lower than the value for the human menisci (40–150 MPa) [16], but in the same range of values reported for similar types of scaffolds, i.e., 0.3 and 0.7 MPa for polyester-PU [60], or polyester-gelatin [61] scaffolds, respectively. Additionally, the material possessed a Shore A hardness of 33.1 ± 3.8, and a glass transition temperature Tg = −34 °C, as measured by DSC, which confirms that the material is soft and flexible.

## 4. Conclusions

In this study, the kinetics of the reactions between primary and secondary alcohol with lysine diisocyanate at 35 °C in the presence of four catalysts, namely MSA, TFA, DMCHA and Sn(Oct.)_2_, were investigated using ^1^H NMR spectroscopy. Among these catalysts, the stannous octoate was found to be the most effective for both primary and secondary alcohols. Additionally, similar reactivities were observed for the primary and secondary isocyanate groups in the presence of these catalysts. The enhanced reactivity of the secondary isocyanate in LDI, compared to that in other isocyanates such as IPDI, can be attributed to the electron-withdrawing effect of the neighboring ester group. Interestingly, DMCHA, a well-known catalyst for the isocyanate/alcohol reaction, was found inactive for the LDI/alcohol reaction, which suggests the presence of acid compounds in LDI. Among the four catalysts studied, Sn(Oct.)_2_ was selected to catalyze the bulk crosslinking of aPδDL_15-3NCO_/glycerol formulation at 35 °C, yielding a crosslinked PU intended to serve as a biocompatible and biodegradable adhesive for meniscus repairment. In those conditions, a gelling time of 18 min was measured by rheology. This fully biobased PU elastomer has an elastic modulus of 519.4 kPa, which is in the same range of tissue adhesives reported in the literature. Additionally, its swelling index measured in phosphate-buffer saline is only 3%, which should avoid swelling of the material in physiological conditions and, thus, subsequent pain associated with an increase in pressure in the knee. While this PU adhesive appears to be a promising candidate as an injectable cartilage adhesive, further studies are required to evaluate the cross-linking in-body environment where water and body fluids may affect the final material properties.

## Figures and Tables

**Table 1:** Volumes of the reagents for each ^1^H NMR experience.

	Volume (μL)									
Experiment	1	2	3	4	5	6	7	8	9	10
**LDI**	117	117	117	117	117	117	117	117	117	117
1-Butanol	111		111		111		111		111	
3-Pentanol		132		132		132		132		132
TFA solution			20	20						
MSA solution					20	20				
Sn Oct solution									20	20
DMCHA solution							20	20		
CDCl_3_	382	361	362	341	362	341	362	341	362	341

**Table 2:** General information of catalysts employed in the screening process.

Catalyst	Formula	LD_50 (rat, oral)_ (mg·kg^−1^) ^a^	pKa ^b^
TFA	* 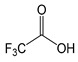 *	500/1000 [47]	0.23/0.26 [48,49]
MSA	* 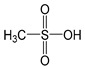 *	648 [50]	−1.9 [51,52]
Sn(Oct.)_2_	* 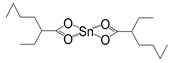 *	5870 [53]	-
DMCHA	* 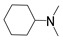 *	348 [54]	10.9 [54]

^a^ LD50: dose at which the substance is lethal for 50% of animals tested (rats). ^b^ pKa: pH at which a half of the considered molecule is 50% protonated.

**Table 3:** Study of the reaction of LDI with 1-Butanol or 3-Pentanol, using TFA, MSA, DMCHA or Sn(Oct.)_2_ catalysts with a NCO/OH/Cat molar ratio of 1/1/0.01.

Exp.	Alcohol	Catalyst 1%_mol vs. Iso_	Conv_alcohol_^a^ (%) t = 60 min	Conv_NCO-I_^b^ (%) t = 60 min	Conv_NCO-II_^c^ (%) t = 60 min	TOF ^d^ (s^−1^)
**1**	1-butanol	none	25	13	12	37
**2**	3-pentanol		17	9	8	23
**3**	1-butanol	TFA	29	9	20	42
**4**	3-pentanol		14	7	7	18
**5**	1-butanol	MSA	37	21	16	44
**6**	3-pentanol		22	15	7	18
**7**	1-butanol	DMCHA	25	6	18	36
**8**	3-pentanol		9	1	8	17
**9**	1-butanol	Sn(Oct.)_2_	96	49	47	1083
**10**	3-pentanol		73	36	37	224

^a^ Determined by ^1^H NMR using Equation (6). The integration of the signal attributed to the protons in α-position to the alcohol (proton K) was monitored: at 3.44 ppm for 1-butanol, and at 3.29 ppm for 3-pentanol. The signal of 1,3,5-trioxane at 4.95 ppm was calibrated to 6H and used as internal standard. ^b^ Determined by ^1^H NMR following the decrease in the signal corresponding to the methylene protons in α to the isocyanate (proton d) at 3.18 ppm, and confirmed by the increase in the signal of the corresponding urethane (proton d’) at 3.0 ppm. The signal of 1,3,5-trioxane at 4.95 ppm was calibrated to 6H and used as internal standard. ^c^ Calculated as the difference between the conversions of the alcohol and the primary isocyanate, according to Equation (7). ^d^ Turnover frequency (TOF) calculated using Equation (1).

## Data Availability

The original contributions presented in this study are included in the article/Supplementary Material. Further inquiries can be directed to the corresponding authors.

## Supplementary Materials

The following supporting information can be downloaded at: https://www.mdpi.com/article/10.3390/polym17182548/s1.
