# Supplementary material for: Catalyst Selection for Body-Temperature Curable Polyurethane Networks from Poly(δ-Decalactone) and Lysine Diisocyanate
Source: Polymers (Basel). 2025 Sep 20;17(18):2548. doi: 10.3390/polym17182548 (PMC12473467; doi:10.3390/polym17182548)
Supplement: Supplementary file 1 [file polymers-17-02548-s001.zip › polymers-3794688-supplementary.pdf]

Supporting Information

Catalyst Selection for Body-Temperature Curable Polyurethane Networks from Poly(δ-decalactone) and Lysine diisocyanate

Marine BOURSIER,[a] Aurelien LEBRUN,[b] Karine PARRA,[b] Sylvain CAILLOL,[a] Claire NEGRELL,*[a] Julien PINAUD*[a]

[a]ICGM, Univ Montpellier, CNRS, ENSCM, Montpellier, France

[b]PAC Chimie Balard Montpellier - UAR2041, CNRS/ENSCM/Université Montpellier, Montpellier, France

*Corresponding authors: claire.negrell@enscm.fr; julien.pinaud@umontpellier.fr

Summary:

Figure S1 ^{1}H NMR spectrum with proton attribution of PδDL15-3OH ($CDCl_3$, 400 Hz, RT) 2

Figure S2 Conversion of the primary (△) and secondary (☐) isocyanates of LDI vs. time with 3-pentanol, Iso I – control (orange △), Iso I + TFA (green △), Iso I + MSA (blue △), Iso I + DMCHA (red △), Iso I + $Sn(Oct)_2$ (black △), Iso II – control (orange ☐), Iso II + TFA (green ☐), Iso II + MSA (blue ☐), Iso II + DMCHA (red ☐), Iso II + $Sn(Oct)_2$ (black ☐). ($CDCl_3$, 400 MHz, RT) 2

Figure S3 Reaction equation of BuOH with IPDI 3

Figure S4 Conversion of the BuOH vs. time for the reaction (35°C) with IPDI for control (orange ●), TFA (green ●) and MSA (blue ●).. 3

Figure S5 Conversion of the primary (▲) and secondary (■) isocyanate of IPDI vs. time with BuOH, Iso I – Control (orange ▲), Iso I – TFA (green ▲), Iso I – MSA (blue ▲), Iso II – Control (orange ■), Iso II – TFA (green ■), Iso II – MSA (blue ■). 3

Figure S6 FTIR spectra and assignments of $P\delta DL_{15\text{-}3OH}$ (blue), LDI (red) and $P\delta DL_{15\text{-}3NCO}$ (green)............ 4

Figure S7 Picture of contact angle measurements performed on A) a glass side and B) the same glass slide coated with the PU material…………………………………………………………………………………………………….5

Figure S8 Tensile stress vs elongation at break for $P\delta DL_{15\text{-}3NCO}$ based elastomer (6 samples)……………..6

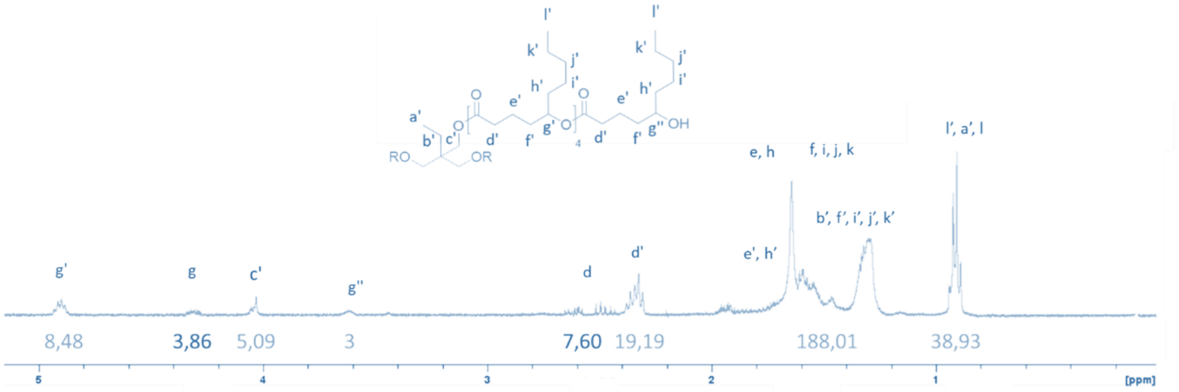

Figure S1 *^{1}H NMR spectrum with proton attribution of PδDL15-3OH ($CDCl_3$, 400 Hz, RT)*

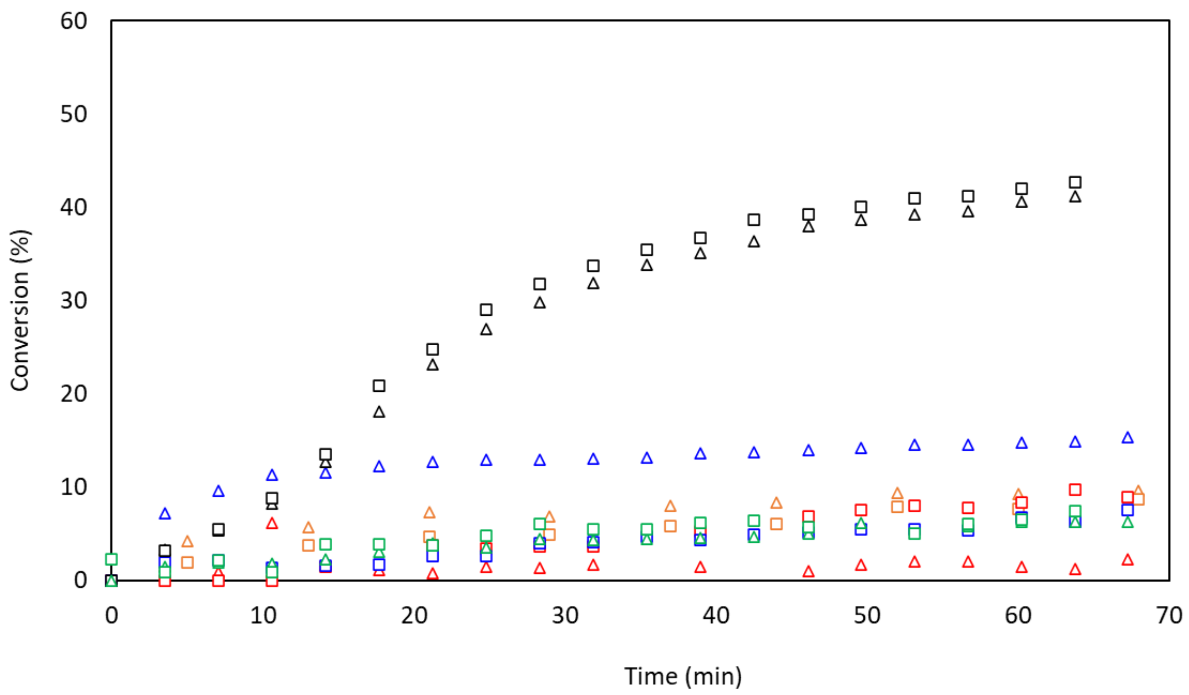

Figure S2 *Conversion of the primary (△) and secondary (□) isocyanates of LDI vs. time with 3-pentanol, Iso I – control (orange △), Iso I + TFA (green △), Iso I + MSA (blue △), Iso I + DMCHA (red △), Iso I + Sn(Oct)$_2$ (black △), Iso II – control (orange □), Iso II + TFA (green □), Iso II + MSA (blue □), Iso II + DMCHA (red □), Iso II + Sn(Oct)$_2$ (black □). ($CDCl_3$, 400 MHz, RT)*

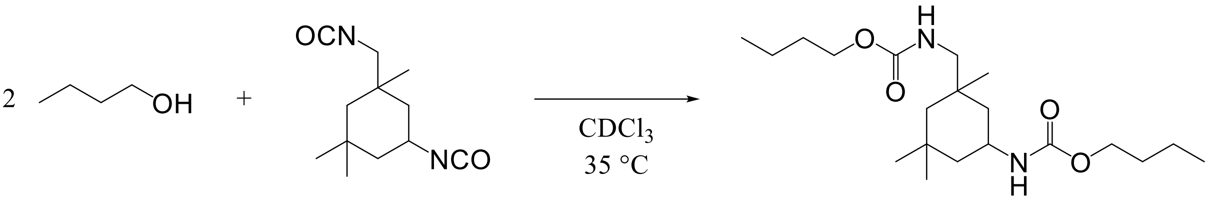

Figure S3 *Reaction equation of BuOH with IPDI*

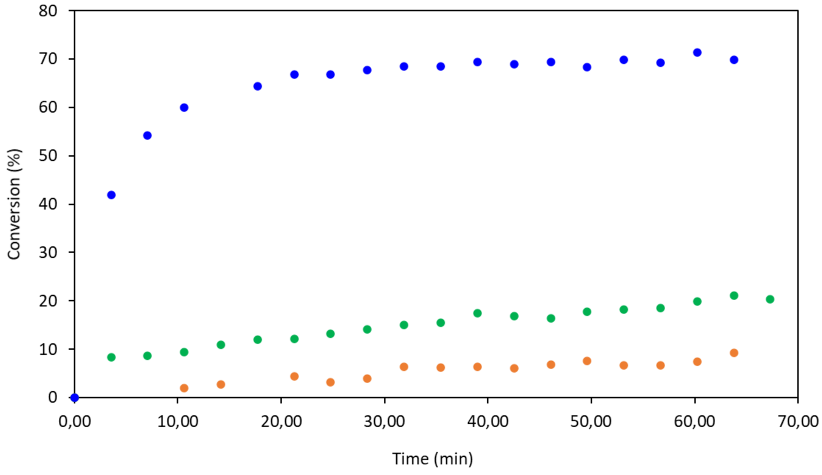

Figure S4 *Conversion of the BuOH vs. time for the reaction (35°C) with IPDI for control (orange), TFA (green) and MSA (blue)*

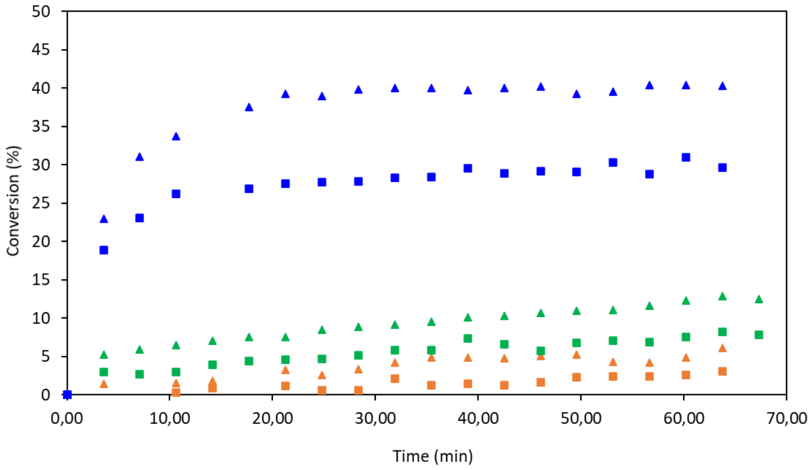

Figure S5 *Conversion of the primary (▲) and secondary (■) isocyanate of IPDI vs. time with BuOH, Iso I – Control (orange ▲), Iso I – TFA (green ▲), Iso I – MSA (blue ▲), Iso II – Control (orange ■), Iso II – TFA (green ■), Iso II – MSA (blue ■).*

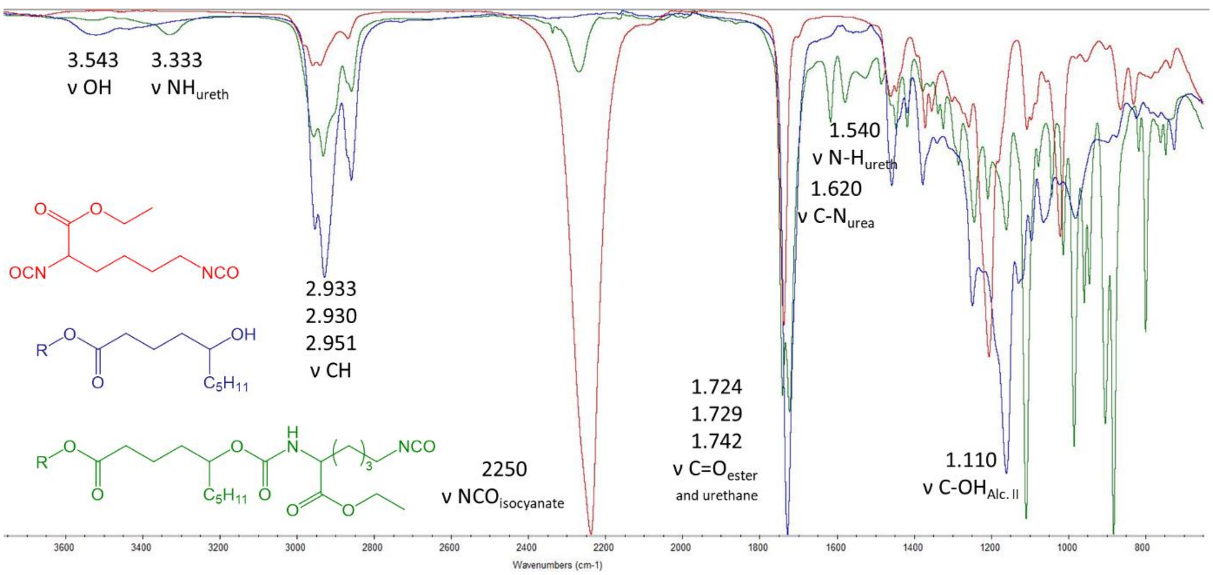

Figure S6 *FTIR spectra and assignments of PδDL$_{15\text{-}3OH}$ (blue), LDI (red) and PδDL$_{15\text{-}3NCO}$ (green)*

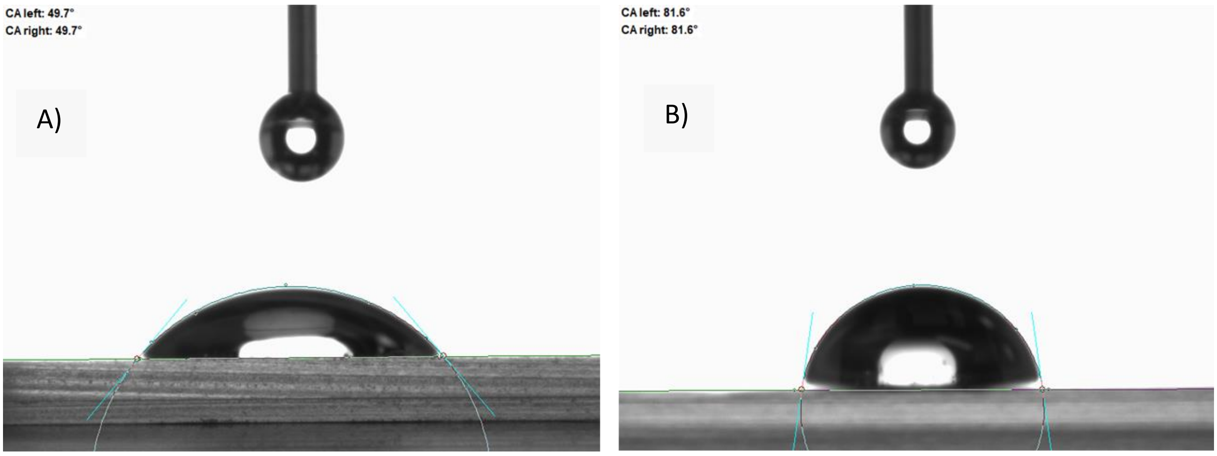

Figure S7 *Picture of contact angle measurements performed on A) a glass side and B) the same glass slide coated with the PU material.*

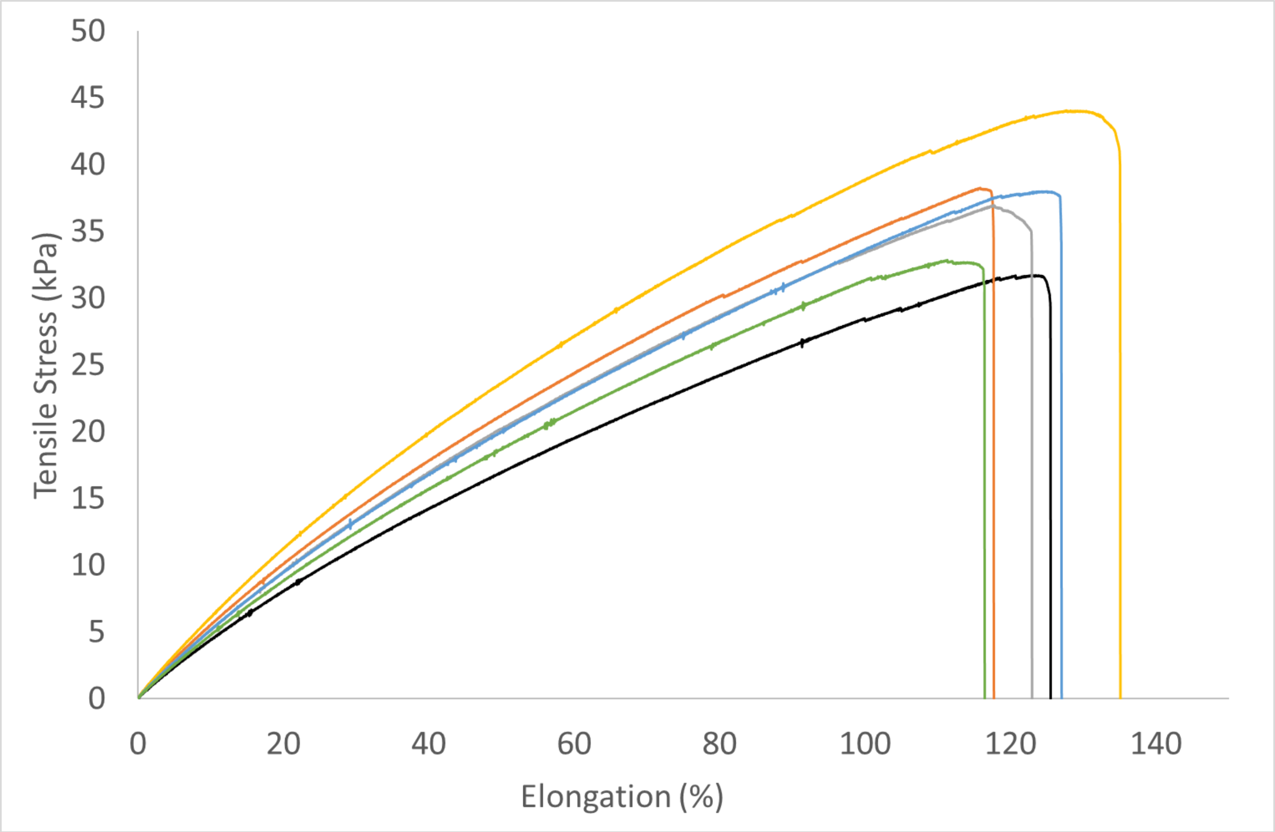

Figure S8 *Tensile stress vs elongation at break for PδDL$_{15\text{-}3NCO}$ based elastomer (6 samples)*
